# The Daunomycin: Biosynthesis, Actions, and the Search for New Solutions to Enhance Production

**DOI:** 10.3390/microorganisms12122639

**Published:** 2024-12-19

**Authors:** Baveesh Pudhuvai, Karel Beneš, Vladislav Čurn, Andrea Bohata, Jana Lencova, Radka Vrzalova, Jan Barta, Vladimir Matha

**Affiliations:** 1 Department of Genetics and Biotechnology, Faculty of Agriculture and Technology, University of South Bohemia in České Budějovice, Studentská 1668, 370 05 České Budějovice, Czech Republic; curn@fzt.jcu.cz; 2 VUAB Pharma A.S, Nemanicka 2722, 370 01 České Budějovice, Czech Republic; benes.karel@hotmail.com (K.B.); vladimir.matha@seznam.cz (V.M.); 3 Department of Plant Production, Faculty of Agriculture and Technology, University of South Bohemia in České Budějovice, Studentská 1668, 370 05 České Budějovice, Czech Republic; lencova@fzt.jcu.cz (J.L.); r.vrzalova@seznam.cz (R.V.); barta@fzt.jcu.cz (J.B.)

**Keywords:** daunomycin, anthracyclines, *Streptomyces*, efflux, autotoxicity, enhancement, oil-based medium

## Abstract

Daunorubicin (DNR) is an anthracycline antibiotic originating from soil-dwelling actinobacteria extensively used to treat malignant tumors. Over the decades, extensive attempts were made to enhance the production of anthracyclines by introducing genetic modifications and mutations in combination with media optimization, but the target production levels remain comparatively low. Developing an appropriate culture medium to maximize the yield of DNR and preventing autotoxicity for the producing organism remains a challenge. Our prospective review sheds light on a method involving perturbation that enhances the precursors to regulate the type II PKS pathway, enhancing cells’ capacity to increase secondary metabolite production. The suggested method also entails the preparation of culture media for the cultivation of *Streptomyces* sp. and enhanced yield of DNR, as well as making it inactive with iron or its reduced forms following efflux from the producer. The iron or iron–DNR complex is encapsulated by oleic acid or lipid micelle layers in the culture media, finally resulting in the generated inactive DNR and the DNR–iron–oil complex. This idea has the potential to protect the producer organism from autotoxicity and prevent the inhibition of metabolite production. The approach of substituting sugar with oil in culture media has a dual role wherein it promotes *Streptomyces* growth by utilizing lipids as an energy source and encapsulating the generated DNR–iron complex in the medium. In this review, we discussed aspects like anthracycline producers, biosynthesis pathways, and gene regulation; side effects of DNR; mechanisms for autotoxicity evasion; and culture media components for the enhancement of DNR production in *Streptomyces* sp. We anticipate that our work will help researchers working with secondary metabolites production and decipher a methodology that would enhance DNR yield and facilitate the extraction of the resulting DNR by lowering costs in large-scale fermentation.

## 1. Introduction

Anthracyclines such as daunomycin are a class of chemotherapeutic compounds derived from soil-dwelling gram-positive actinobacteria that have been used as anticancer medication agents [1]. They have been widely utilized to treat leukemia and cancer in adults and pediatrics since their discovery in *Streptomyces peucetius* FI 1683 in the 1960s [2,3,4,5,6]. The term “Anthracyclines” was introduced to denote the color (red to yellow–red optical dyes) of the chemical derivatives 7,8,9,10-tetrahydro-5,12-naphtacenoquinones [7,8]. The anthracyclines are tetracyclic aromatic polyketides that are produced by the PKS-II (type II polyketide synthase) pathway and are structurally composed of an anthraquinone (aglycone) moiety and an amino sugar (carbohydrate unit) at the C7 or C10 or at both positions. The absence of sugar at C10 is substituted by a carbo-methoxy or hydroxyl group through processes like glycosylation and hydroxylation [1,9,10].

**Figure 1:**
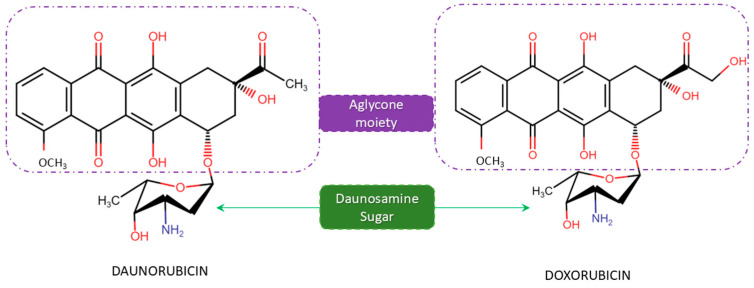
Structure of daunorubicin (DNR) and doxorubicin (DOX) with the aglycone sugar moieties.

Daunorubicin (DNR), also called daunomycin, is an anthracycline antibiotic that has been extensively employed in treating malignant tumors, ovarian cancer, breast cancer and AML (acute myeloid leukemia) since its discovery in 1964 from *Streptomyces peucetius* [11,12,13]. DNR was initially derived from *Streptomyces peucetius* FI 1762-B-101 and was found to possess antitumor activities. During the same period, French researchers isolated a similar substance from *Streptomyces ceoruleorubidus* J566, naming it rubidomycin [14]. The successful characterization of the compound and its clinal-use case has led to the development of several hundreds of anthracycline analogues through synthetic chemistry and modified bacteria [15]. The latter research in 1969 on a mutant of *S. peucetius* ATCC 29050 has led to the identification and isolation of a homologue (related compound) named doxorubicin (DOX) in *S. peucetius* subsp. *caesius* ATCC 27952 with better efficiency in solid tumors [16]. The DNR and DOX share tetracyclic aglycone and daunosamine sugar moieties but differ in their side chains, where DOX terminates with primary alcohol and DNR with a methyl group, as shown in Figure 1.

The clinical use of DOX and DNR was hampered due to increased concerns and deaths due to cardiotoxicity and the development of resistance in tumor cells [5,15]. Despite the demonstrated toxicity of daunomycin in Guinea pigs, rats, and humans [17,18,19], the cumulatively reduced dosages administered during chemotherapy allow the mitigation of these risks [20,21,22]. A minor structural modification of anthracyclines significantly affected bioactivity, which had driven chemists in the 1970s to synthesize anthracycline derivatives with reduced toxicity [10]. The interest and progress in synthesis pathway engineering (synthetic and semi-synthetic via site-directed mutations and gene alterations) of anthracyclines and their analogues were carried out after the 1990s due to their exciting catalyzing properties [8]. The quest to find a better alternative with reduced toxicity has led to thousands of analogues with many substitution reactions in the anthraquinone moiety (tetracyclic structure) [5]. Out of which, currently, six semi-synthetic derivatives, including DOX, idarubicin, epirubicin, pirarubicin, and valrubicin, are under clinical use. The class of anthracyclines and their derivatives, including doxorubicin, epirubicin, daunorubicin, and idarubicin, are the most potent anticancer drugs ever discovered, having the ability to both alter mitochondrial dynamics by intercalating with DNA helix and cause cytotoxicity. These drugs cause accelerated senescence in tumor cells via inducing apoptosis, DNA alkylation and damage, autophagy, activating MAPK (mitogen-activated proteins kinases), promoting antimetabolites, inhibiting topoisomerases, and other mechanisms that result in cell death or cell cycle arrest [23,24,25,26,27,28].

Over the past decades, numerous efforts have been made to generate anthracyclines through multiple initiatives, which have led to the creation of several strains with genetic modifications, mutations, and changes to culture conditions and media. Nevertheless, despite the accomplishment of numerous intricate investigations and genetic modifications, the desired output production numbers by fermentation have yet to be attained. Engineering suitable culture media to sufficiently achieve the enhanced production of DNR/DOX and make them inactive to evade the self-toxicity to the producer organism remains void. The alteration of strain for enhanced production and culture media conditions in order to entrap the produced DNR/DOX compounds in media and turning them non-toxic for the producer proves an ideal strategy. Considering the complex gene functions and metabolic intricacies in the PKS-II pathway, the alterations towards the efflux systems, metabolic pathway inducers, and interaction of DNR/DOX with other elements and substances should be considered to design hypothetical culture media and conditions.

In this review, we have discussed several aspects of the production of DNR/DOX by *Streptomyces*, their activity, biosynthesis, gene regulation, toxicity, and how the culture media could be designed for production enhancement based on the interaction of daunomycin with iron and oil and self-resistance properties of the producer organism. Our prospective idea of strain development and culture media optimization to trap the produced DNR in media using oil and iron would be a beneficial method to improve DNR production at an industrial scale. We anticipate that this review benefits the investigators in the microbiology, bioprocessing, therapeutic, and industrial biotechnology fields to enhance DNR production with reduced expenses at a commercial level.

## 2. The Anthracycline Producers and Daunomycin Synthesis

Streptomycetes are Gram-positive bacteria well known for synthesizing various secondary metabolites such as antibiotics, enzymes, and pigments [29,30]. They contain a multitude of biosynthetic gene clusters (BCGs) and transcription units in their genome, which are responsible for the production of secondary metabolites [31]. A substantial proportion of known antibiotics are derived from the organisms of the genus *Streptomyces*. To date, more than 500 naturally occurring anthracyclines have been isolated from *Streptomyces* spp., which are widely considered medically important [32,33]. *Streptomyces ceoruleorubidus* is a potentially important bacteria that synthesizes antifungal, antibacterial, immunosuppressive, and antitumor (doxorubicin and daunorubicin) compounds [34,35,36]. The production of secondary metabolites occurs through two phases, the trophophase (normal growth phase) followed by the idiophase (capacity to produce metabolites), where, at times, both phases can be regulated, overlapped, and changed with the alterations in media and growth conditions [5]. Enhancing secondary metabolite production can be possible with the development of cell resistance as the compounds produced are autotoxic. However, during any selection event over the past decades, the focus on improved resistance did not impact or lead to enhanced production. This led to the works on biosynthetic gene cluster alterations and expression enhancement of activator genes, transcription factors, and increased mutations in promotor genes [5,37].

### 2.1. Biosynthetic Gene Clusters (BGCs)

The importance and biosynthesis of daunorubicin and its gene clusters have been characterized by two BGCs in different strains [38,39]. Most BGCs share homologous genes encoding monofunctional enzymes to assemble aglycone units. The BGCs for DNR (daunorubicin) and DOX (doxorubicin) were sequenced (40 kb) in *Streptomyces peucetius* ATCC 27952 [40]. The distinctive characteristics among the BCGs include a high abundance of glycosyl transferases, gene sets involved in deoxysugar production, and a repertoire of tailoring genes for secondary metabolites. The DNR/DXR biosynthesis is completed in four steps: (A) formation of aglycone (ε-rhodomycinone), (B) formation of an active sugar moiety (thymidine diphosphate daunosamine), (C) glycosylation of ε-rhodomycinone, and (D) post polyketide modifications (decarboxylation, methylation and hydroxylation) [28,38,41].

#### 2.1.1. Formation of ε-Rhodomycinone

**Figure 2:**
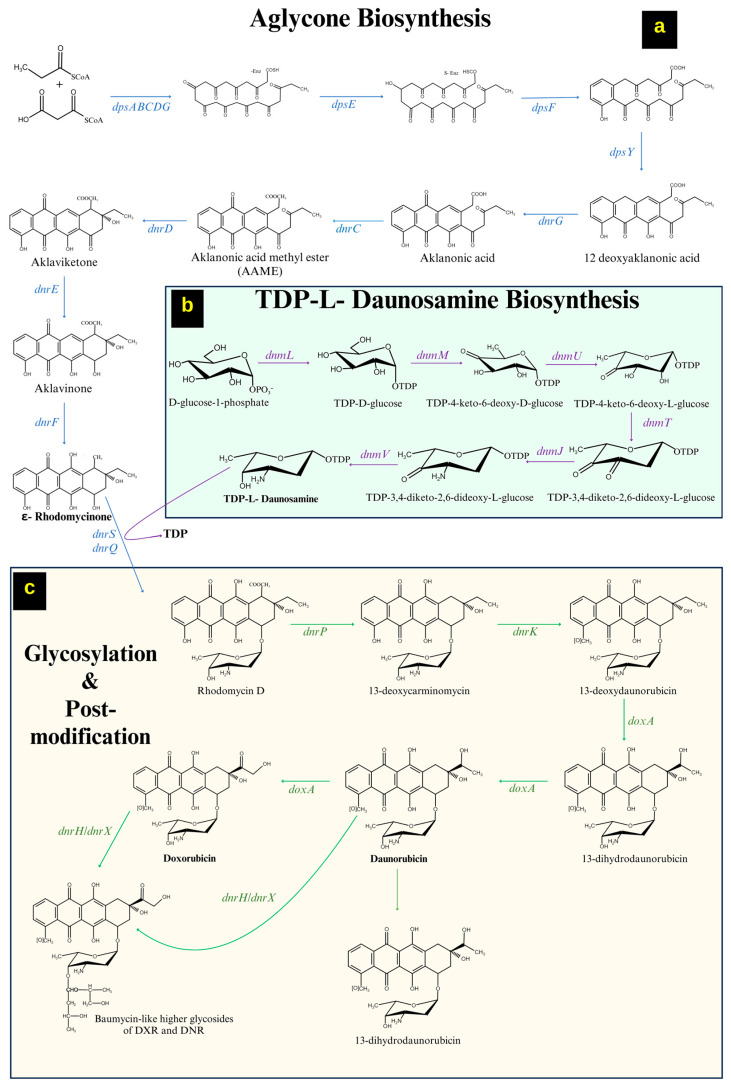
Biosynthesis pathway and involved genes of daunorubicin (DNR) and doxorubicin (DOX) in *Streptomyces* with (**a**) the aglycone moiety synthesis, (**b**) the sugar moiety, and (**c**) the glycosylation and post-modification steps in DNR/DOX synthesis.

The biosynthesis of DNR starts with the formation of aglycone ε-rhodomycinone, which is the important intermediate, synthesized by the PKS-II synthase by the genes *dpsA*, *dpsB*, *dpsC*, *dpsD*, *dpsE*, *dpsF*, *dpsG,* and *dpsY*. The nine malonyl-CoA units with a propionyl CoA starter unit undergo serial condensations to form a 21-carbon decaketide, where the multiple reactions are facilitated through the polyketide synthase enzymes. The enzymes are produced by the genes *dpsA* (3-oxoacyl ACP synthase), *dpsB* and *dpsC* (ketosynthases), *dpsD* (acyltransferase), and *dpsG* (acyl carrier protein) to form a decaketide compound [42]. The *dpsE* (ketoreductase) carries out the ketoreduction of the decaketide, followed by an aldol condensation and ring cyclization (3 steps) through catalyzing enzymes produced by *dpsF*, and *dpsY* forms a 12-deoxy alkanoic acid [43]. The intermediate undergoes a *dnrG* (monooxygenase)-mediated reaction, which adds a keto group to form alkalonic acid and is further transformed to aklaviketone by *dnrC* (alkanoic acid-S-adenosyl-1-methionine methyl ester transferase)—a homodimeric protein [38,44]. The formed aklaviketone intermediate undergoes cyclization by *dnrD* (alkanoic acid methyl ester cyclase) to form a 7-oxo moiety, which is further reduced to a hydroxy group of aglycone ε-rhodomycinone by *dnrH* (aklaviketone reductase) and *dnrF* (hydroxylase) [44]. The biosynthesis of aglycone ε-rhodomycinone, including the genes involved, is shown in Figure 2a.

#### 2.1.2. Formation of a Sugar Moiety (Thymidine Diphosphate-L-Daunosamine)

The biosynthesis of dTDP-_L_-Daunosamine is initiated from D-glucose-1-phosphate, which is carried out by the BGC, including seven genes *dnmL*, *dnmM*, *dnmU*, *dnmT, dnmJ,* and *dnmV* [42]. The *dnmL* (transferase) and *dnmM* (dehydratase) catalyze the reaction to generate the intermediate TKDG (thymidine diphosphate-4-6-deoxy-D-glucose). The epimerase produced by *dnmU* converts TKDG to TKLG (thymidine diphosphate-4-6-deoxy-L-glucose) through epimerization [45]. The *dnmT* (hydratase) and *dnmJ* (aminotransferase) facilitate the addition of a keto and an amino group at the C-3 position of the TKLG intermediate. The *dnmV* (ketoreductase) reduces the ketone to the hydroxyl group at the C-4 position to produce dTDP-_L_-Daunosamine [46]. The biosynthesis of an active sugar moiety (Thymidine diphosphate-L-daunosamine), including the genes involved, is shown in Figure 2b.

#### 2.1.3. Tailoring Reactions/Modifications in DNR/DOX Biosynthesis

The ε-rhodomycinone undergoes glycosylation with dTDP-_L_-Daunosamine in the presence of enzyme from *dnrS/dnrQ* to generate rhodomycin D. The *dnrP* (esterase) converts rhodomycin D to 13-deoxy-carminomycine, which undergoes an *O*-methylation by *dnrK* (methyltransferase) to generate 13-deoxy-daunorubicin [47]. The intermediate undergoes C-13 oxidation by *DoxA* (cytochrome P450 enzyme) in two steps to produce 13-dihydro daunorubicin and DNR (daunorubicin) [48]. Daunorubicin is hydroxylated later at the C-14 position through *DoxA* to form DOX (doxorubicin) [42,49]. The biosynthesis of DNR and DOX, including the genes involved, is shown in Figure 2c.

### 2.2. Gene Regulation in DNR/DOX Biosynthesis

The BGC responsible for the biosynthesis of polyketide and sugar moieties in DNR/DOX also includes the regulatory genes for the initiation, regulation, and termination of the entire synthesis pathway. The production pathway is regulated by the genes including, *dnrO*, *dnrN,* and *dnrI*, and the transcription factors, where *dnrO* holds a significant importance in initiating the pathway. The *dnrO* encodes a DNA helix binding domain, which is a key transcriptional regulator that activates the *dnrN* transcriptional activator, which finally leads to the activation of *dnrI*. The *dnrI* encoding enzyme binds to several polyketide synthases and facilitates the activation of efflux regulatory genes and initiation of DNR biosynthesis. The BGC also includes a transcriptional repressor *drrD*/*dnrW*, which promotes transcriptional control by coherent feed-forward loop, self-resistance, and feedback regulation [42,50]. The *drrD*/*dnrW* regulates the master transcription factor *dnrI,* which is crucial for the DNR/DOX biosynthesis. Deleting *dauW* (ortholog of *drrD*/*dnrW* in *S. ceoruleorubidus*) has increased the production of DNR by eight folds [51].

The maintenance of the produced DNR requires regulation inside the producer organism as the compound exhibits toxicity by intercalating with cellular DNA and eventually leading to cell death. The regulation of the lethal concentrations of produced DNR inside the cell is conferred by the *drrAB* locus—includes the drrA and drrB proteins necessary for the efflux of the finished product [50,52]. The expression and function of drrA and drrB are interdependent on each other at an ATP-driven pump, where drrA is a peripheral membrane protein acting as an energy-transducing unit inside the cell when bound to the ATP in a DOX-dependent manner and drrB is the internal protein with hydrophobicity and helps in the efflux of produced DNR/DOX [53,54]. A mutant strain without the *drrAB* has exhibited a decline in DNR production and resulted in cell death, and overexpression of *drrAB* has resulted in the overproduction of DNR and promoted self-resistance [55]. Thus, the self-resistance genes also indirectly affect the biosynthetic pathway in DNR/DOX production [56]. Another resistance gene is *drrC*, which functions in the presence of ATP and DNR by binding to the DNR intercalated DNA and propelling it outside of the cell. This self-resistance gene maintains cell viability and regulates the lethal concentrations of DNR in a dependent manner, which relies on *dnrN* and *dnrI* in the biosynthetic pathway [47].

The entire pathway and its regulation decide the fate of DNR/DOX quantity production in *Streptomyces* spp. Thus, over the past decades, researchers have considered engineering the genes involved in the biosynthesis of aglycone, sugar moiety, tailoring reactions, transcriptional factors, transcriptional repressor, and self-resistance to improve DNR/DOX production at an industrial level for commercial uses in cancer medication. The present techniques of modifying genes to enhance the production of DNR/DOX are not effective due to the complex cellular enzymatic reactions involved. These approaches have not provided a clear understanding of the entire mechanism and could not contribute significantly to improvements in metabolite production.

## 3. Daunomycin Mode of Action

Since their discovery, the DNR and DOX have been extensively employed for treating solid tumors but have faced significant drawdown due to their toxic properties. Anthracyclines enter cells through cation transport and passive diffusion, eventually leading to alterations in the proteasome and nucleosome [57].

### 3.1. DNA Intercalation

Anthracyclines exhibit a strong affinity for DNA by inserting their aglycone moieties between the base pairs, causing the separation of the existing base pairs, and positioning their sugar components in the minor groove of the DNA [22,58]. DNR and DOX have a preferential ability to bind to DNA at GC base pairs of both mitochondrial and nuclear DNA by establishing hydrogen bonding between the hydroxyl group on the C-9 position at aglycone moiety and N2, N3 of guanine [59,60,61]. This inhibits cellular DNA transcription, replication, recombination, and repair, which creates torsional stress. The torsional stress alters the structure (disassociation of H2A/H2B dimers from histone core) and dynamics of nucleosomes [62,63]. The histone eviction caused by DOX/DNR (in H3 due to rich GC base pairs), majorly due to the sugar moiety binding to DNA, critically causes chromatin damage, which leads to epigenomic aberrations and transcriptional alterations [57,64].

### 3.2. Topoisomerase II (Topo II) Poisoning

The topoisomerase II (topo II) induces double-stranded breaks (DSBs), releases torsional stress and re-ligates the DNA breaks, ensuring the proper DNA transcription, replication, and repair [65]. Anthracyline interacts with the topo II enzyme to form an anthracycline–topoisomerase–DNA quarternary complex. It induces irreversible DNA damage by preventing the regeneration of phosphodiester bonds between the DNA strands [57]. DNR/DOX intercalates the topo II DNA with their cyclohexane ring A in aglycone moiety and 4-methoxy group in sugar moiety. The changes in the functionality of topo II to a DNA nuclease generate genomic instability, activation of DNA damage response, and TP53 pathways, eventually leading to cell death [15]. In mammals, the topo II enzyme is distinguished into isoforms topo IIα (generate replication forks during mitosis in actively diving cells) and topo IIβ (expressed in most cell types devoid proliferation status), where the DOX interacts with topo IIβ in cardiomyocytes, leading to cardiotoxicity [66,67].

### 3.3. Formation of DNA Adducts

Anthracyclines form DNA adducts between the two strands through covalent and hydrogen bonds with aglycone and sugar moieties, respectively. The DOX-DNA covalent bond in the cancerous cell is facilitated by the cellular formaldehyde; produced due to free radicle reactions with polyamines and lipids it is responsible for the block in transcription, DSBs, and replication [22,68]. In vitro studies using DOX by pre-activated formaldehyde resulted in the formation of transcriptional blocks through the formation of inter-strand adduct (G-DOX-G cross-linking), inhibiting the transcription process [69]. The treatment of mice cancer cell lines with DOX leads to the disruption of the replication process and cell cycle arrest through the blocks in [8H]-thymidine [70,71]. The investigations involving DOX and DOX-formaldehyde conjugate on colorectal cancer cell lines for DNA repair mechanisms resulted in DNA adduct-induced damage. The studies also prove the damage (apoptosis) caused by DOX-DNA adducts is independent and does not rely on the topo II activity [72,73,74].

## 4. Side Effects of DNR/DOX

Over the past decades, DNR/DOX has been significantly used in cancer treatment; however, its application is associated with adverse effects predominantly affecting bone marrow and cardiac muscle, resulting in bone marrow suppression and cardiotoxicity [15]. Their associated side effects on healthy cells during treatment adversely affect their functionality, including acute and reversible chemotherapy-related symptoms such as nausea, vomiting, diarrhea, stomatitis, mucositis, alopecia, gastrointestinal problems, rash, and bone marrow suppression [57]. The long-term effects include cardiotoxicity, nephrotoxicity, gonadotoxicity, and several therapy-related malignancies, which impact the patient’s quality of life and severely limit the usage of anthracyclines. Factors like dosage, treatment length, and the patient’s individual risk factors determine the possibility of developing anthracycline-related adverse effects.

### 4.1. Cardiotoxicity

Cardiotoxicity is a well-documented adverse effect of anthracycline chemotherapy encompassing both acute and chronic detrimental impacts on the heart, ranging from myocardial changes, impaired contraction ability, cardiomyopathy, arrhythmias, and heart failure necessitating heart transplantation [75,76]. The mechanisms involving the DNR/DOX-induced cardiotoxicity are due to the inhibition of topoisomerase [77], mitochondrial dysfunction (membrane permeability and transcription enzymes) [78,79], iron ion metabolism, imbalance in calcium homeostasis in cardiac muscles [80], oxidative stress and ROS generation [81], loss of ATP production [82], and cell apoptotic pathways [80,81,83]. The molecular mechanisms explaining the detailed functioning effects of individual DNR/DOX-induced pathways regarding mitochondrial activity and apoptosis were explained here [79,84,85].

The cardiomyocytes hold negligible amounts of active free iron, and most iron is bound to cellular proteins. The DOX/DNR possesses a strong affinity for iron and disrupts the iron hemostasis in the cells through a redox reaction, reducing the cellular iron and cyclically forming the DOX-Fe complexes between Fe^2+^ and Fe^3+^ [83]. Under abundant iron, daunomycin increases cellular ROS (reactive oxygen species) and induces oxidative stress in cardiomyocytes [79,86].

The prevention of anthracycline-induced cardiotoxicity involves the co-administration of cardio-protectant compounds like dexrazoxane, neuregulin, β-blockers (carvedilol and nebivolol), aldosterone antagonists, atorvastatin, angiotensin receptor blockers (ARBs), ascorbic acid, and sodium-glucose transport protein-2 inhibitors (SGLT-2) [87,88]. The iron chelator dexrazoxane reduces anthracycline-dependent ROS generation, oxidative stress, and DNA double-strand breaks. It has significant clinical efficacy, decreasing cardiac toxicity without reducing anthracycline activity or enhancing secondary malignancies.

### 4.2. Redox Mechanisms and Oxidative Stress

Anthracyclines cause apoptosis in cells through alteration of the iron-dependent lipid peroxidation. The iron levels increase in the cells through interaction with iron regulation proteins (IRP1,2). The process is mediated by glutathione peroxidase 4 (GP4), where DOX-Fe^2+^ and DOX-Fe^3+^ adducts are formed and cause the accumulation of lipid-based reactive oxygen species (ROS) [89]. The DOX downregulates the GP4 activity and interacts with genomic and mitochondrial DNA, accumulating iron-DOX adducts and inhibiting the *ABCB8* efflux transporter pump. The inhibition or downregulation of the *ABCB8* efflux transporter increases DOX-induced toxicity, ROS levels, and cardiomyocyte apoptosis [90].

### 4.3. ROS Alleviation and Mitochondrial Dysfunction

Cardiomyocytes have a greater number of mitochondria than regular cells to obtain more energy (ATP) for the contraction function. The DOX accumulation in mitochondria is significantly higher than in the cytosol, which causes DOX-induced mitochondrial impairment to increase ROS in cells [91]. An increase in ROS, a peculiar anthracycline toxicity condition, leads to deformity in cell organelles and membranes and induces cell death. DOX stimulates the generation of superoxide anions in cells in a dose-dependent manner. NADPH mediates the cyclic process: cytochrome P-450 reductase, which includes the transfer of electrons from NADPH to DOX to convert to semi-quinone (DOX-SQ), eventually forming an O_2_ molecule, superoxide anion (O_2_^−^) and a DOX molecule. The superoxide dismutase (SOD) converts superoxide anion to hydrogen peroxide (H_2_O_2_), which then undergoes the Fenton reaction to produce hydroxyl radicals [92]. The DOX alters the ROS production process through interferences in the electron transport system [92,93]. DOX exhibits a high affinity towards cardiolipin in the mitochondrial membrane, transforming the cardiolipin’s attachment ability for cytochrome c and other mitochondrial proteins and altering the normal function [94].

### 4.4. Lipid Dysfunction and Cell Membrane Alterations

DOX can disrupt the lipid organization in the cell, where the interaction with the cell and mitochondrial membrane is high. The localization of DOX in the mitochondria enables it to interact with the inner mitochondrial membrane due to lipid peroxidation, and the resultant lipid aglycone is hard to diffuse out of the membrane to the cytosol. Especially in cardiomyocytes, the mitochondrial dysfunction leading to the proteotoxic burden is due to this DOX lipid interaction [95,96]. Dox hinders the activity of the phosphatidylserine decarboxylase enzyme (catalytic enzyme for phosphatidylserine to phosphatidylethanolamine), a crucial element of cell membranes, thus leading to cellular membrane dysfunctioning [97].

## 5. Self-Resistance in Microbial Factories/Non-Target Species

As discussed in Section 3, the DNR/DOX compounds interact with DNA and inhibit topo II, leading to DNA damage. The microbial cell factories of antibiotics, anthracyclines, and related cytotoxic compounds like filamentous *actinobacteria* are programmed to deal with the cytotoxic compounds made by them [98,99]. These resistance mechanisms include the expression of resistance genes, efflux systems to pump out anthracyclines, the inactivation of anthracyclines through enzymatic modifications, and interaction with other metal elements.

### 5.1. Resistance Genes

The self-resistance developed by the bacteria through the expression of resistance genes is a prerequisite to its survival against the produced toxic (DNA intercalating majorly) compounds. Similar to the antibiotic-pathway-synthesizing genes on BGCs, the resistance genes are also encoded in the BGCs, which initiate the process of self-resistance through time–space coordinated expression or intermediate-dependent (compound-produced) expression [100]. The resistance mechanisms are variable according to BGCs or product type and include target protection, compound inactivation, modification, sequestration, and efflux.

In *Streptomyces peucetius*, the genes encoding resistance for DNR/DOX are *drrA*, *drrB*, and *drrC* unraveled when expressed in *E. coli* and *S. lividans.* The drrA and drrB proteins act as drug-efflux complexes produced during the idiophase, while the drrC is produced earlier and facilitates the efflux through drug binding [52,53,101].

### 5.2. Efflux Pumps

Efflux pumps play a pivotal role in conferring multidrug resistance in bacteria by facilitating the expulsion of toxic compounds either produced by the organism or acquired from the external environment [102]. They are key components of the cell membrane that regulate the internal cellular concentrations of toxic chemicals and elements (metal ions) through extrusion and inhibit compounds’ re-entry to evade toxicity [103,104]. The efflux pumps utilize energy by hydrolyzing ATP and can use the electrochemical or ionic gradient to efflux the toxic compounds. The efflux systems found in bacterial cells are categorized into six families: ABC (ATP-binding cassette), MATE (multidrug and toxic compound extrusion), PACE (proteo-bacterial antimicrobial compound efflux), MFS (major facilitator superfamily), SMR (small multidrug resistance family), and RND (resistance nodulation cell division) [105]. These efflux pumps comprise transmembrane protein helices facilitating the translocation of secondary metabolites outside the producer organisms [106]. However, despite their varied structural differences, substrate redundancy is prevalent across all the efflux pump families. The DOX/DNR is extruded out by the AbeM efflux pump of the MATE family (using antiporters H^+^ and Na^+^) in *Acinetobacter baumannii,* whereas the ABC pumps (generally hydrolyze ATP) perform the extrusion in *Streptomyces* spp. [107,108]. The ABC (ATP-binding cassette) pumps constitute the most prominent protein families and are widely present in all living organisms, facilitating the import and export of chemical substances based on their structural architecture and folding [109]. The ABC efflux pumps in bacteria use energy by hydrolyzing ATP and translocating various chemical compounds like sterols, secondary metabolites, and lipids across the membrane through 12 transmembrane domains (TMDs) and two nucleotide-binding domains (NBDs) [55,110]. The TBDs aid in substrate binding, whereas the NBDs carry the translocation of compounds hydrolyzing ATP. The detailed mechanism and structural diversity of ABC pumps and their activity depending on the arrangement of helices, loops, and protein domains are reviewed by Thomas et al. (2024) [109]. The *drrAB* transporter system encodes for the efflux of DNR/DOX in *S. peucetius*, where *drrA* (peripheral membrane protein) binds to ATP, and *drrB* (hydrophobic membrane protein) enables the translocation acting as a resistance mechanism. The subcloning of these *drrAB* genes in *E. coli* resulted in similar expression [111]. Several follow-up studies conferred the resistance mechanism of the *drrAB* transporter system and the co-dependence of both proteins in efflux activity [112,113]. A recent study by Dong et al. (2024) conducted on an ABC transporter in *Streptomyces ceoruleorubidus* yielded significant findings, indicating that the *drrAB* genes of the DNR BGC facilitate the efflux of excess DNR/DOX within the cell. Additionally, the two-component ABC transporters, encoded by *drrAB2* and *drrAB3* and situated outside the cluster, regulated by the TetR family regulator *drrR1*, were identified as playing a complementary role in the efflux of daunorubicin in *S. coeruleorubidus* in response to the intracellular accumulation of daunorubicin [114].

### 5.3. Inactivation of Drug by Enzymatic Reaction

Resistance mechanisms to evade autotoxicity in microorganisms also include inactivating or modifying the produced metabolites or antibiotics through enzyme activity. Activating the repressor gene *dnrH* in *S. peucetius* carries out the glycosylation reaction of the daunosamine sugar to baumycin-like glycosides, thereby preventing the formation of DNR/DOX [115]. Similarly, the *doxA* gene encoding the cytochrome P450 oxidase, crucial for three oxidation steps in DNR/DOX development, gets downregulated by the excess concentration of produced daunorubicin inside the *Streptomyces* sp. [48,49]. A detoxification strategy of *Streptomyces* by reducing the DOX to 7-deoxydoxorubicinolone via deglycosylation using NADH, ubiquinone oxidoreductases, was reported [116]. Thus, employing the activation of products through enzymes aids in the inhibition of the intercalation of DNA and evades autotoxicity in the producer organisms.

### 5.4. Alteration of Drug Targets

Conferring to resistance towards self-toxicity in microorganisms also involves altering the drug targets. Such modifications inhibit the interaction of produced metabolites—cellular components like ribosomes, DNA, and topoisomerases [50]. Upregulation of genes responsible for methylation for DNA and topo II alteration in response to evade self-toxicity from produced DNR/DOX is a resistance mechanism exhibited by *Streptomyces* spp. Thus, deciphering such systems and enhancing their activity can improve production by the strain in industrial settings [50,117].

## 6. Interaction of DNR/DOX with Iron

Daunomycin is the chelator of iron, where iron forms (Fe^2+^ and Fe^3+^) bind to specific functional groups of anthraquinone moiety and form stable complexes [118]. The quinone group at position five and hydroxy group at position six on the aglycone part of DNR act as the binding sites for iron by donating electrons. The DNR also has a side chain with hydroxyl groups, which can donate a lone pair of electrons and bind to iron [118]. Both ferrous (Fe^2+^) and ferric (Fe^3+^) forms of iron bind to daunomycin, where Fe^2+^ is highly reactive and readily participates in redox cycling and alters between ionic states and Fe^3+^ is less reactive and forms stable complexes [119,120]. This stabilization activity can be employed for therapeutic purposes. The first tri-ferric doxorubicin compound, named Quelamycin, a metallic derivative of the Adriamycin prepared, was through chelation in the presence of Fe (III) [121]. The compound has been reported to be highly stable in phase I clinical trials and P 388 leukemia cells, where the cytosolic components do not degrade the compound. It also inhibits the free flow of electrons from NADH to oxygen molecules in cells [120,122,123]. The bond strength of the iron–DNR complex is high. The chelation activity can be reversible or disassociated in high acidic pH (lower) conditions and the presence of iron-binding compounds like transferrin and ferritin.

## 7. Interaction of DNR/DOX with Oil

The anthracycline compounds daunorubicin and idarubicin are lipophilic and their interaction mechanisms with the lipids are studied using various experiments [124,125,126]. Oils and oleic acids, being non-polar, bind to the hydrophobic regions on the anthraquinone moiety, often used in therapeutic formulation. The liposome-associated doxorubicin was reported to have reduced systemic and cardiotoxicity in clinical trials for humans and mice [127,128,129]. The daunorubicin is encapsulated by the liposomes (phospholipid vesicles) and exploited for drug delivery mechanisms [130,131]. The interactions and their practical implications are clearly reviewed here [132].

## 8. Culture Media for Metabolites Production in *Streptomyces* spp.

The production of antibiotics at a large scale is a combinatorial effect which relies upon the strain efficiency, ability to utilize the available nutrients, physical conditions, and productivity of the metabolites. The primary nutrients like carbon, nitrogen, phosphorus, and minor mineral elements remain the major constituents of the culturing media responsible for the growth and production of necessary chemical compounds in *Streptomyces* spp. Carbon serves as a prominent energy source, nitrogen is responsible for cell growth and metabolism, and phosphates assist in the production of metabolites [131]. To date, many investigations over the decades have concentrated on improving secondary metabolites using strain engineering via genetic alterations. However, the culmination of improved levels of metabolite production through extensive genetic research remains unpromising due to the intricate metabolic mechanisms involved, as reviewed in the biosynthesis and gene regulation sections. Additional investigations employing modifications in media have the potential to result in more streamlined and economical techniques for manufacturing daunomycin and other crucial antibiotics in *Streptomyces* spp.

The DNR/DOX compounds are produced in the late growth phase through a multitude of enzymatic reactions by *Streptomyces* spp., utilizing nutrients [133,134]. The host cells synthesizing the secondary metabolites in nature (µg/L) are not sufficient to achieve the harvest at the desired quantities (g/L) on an industrial fermentation scale [135]. So, the efficient native strains are screened and improved through metabolic engineering (contemporary), mutations (traditional), and selections. However, the highest yields are achieved by combining several approaches in strain development, suitable culture media composition, and well-optimized fermentation conditions. The complete genomic sequencing of the model actinomycete *Streptomyces coelicolor* revealed the presence of multiple genes in the *Streptomyces* genome that can break down complex carbohydrates and proteins [136]. This facilitates utilizing various carbon and nitrogen sources to optimize culture media. The production of metabolites is also linked to factors like nutrients available in culture media and fermentation conditions (temperature, light, oxygen, and pH) [134,135]. The optimization of media and the source of nutrients remained a major variable factor in the growth of strains and the production of metabolites for several decades.

### 8.1. Carbon Source

Glucose or sugars are the most often utilized carbon sources in industrial fermentation due to their low cost and high availability, even though they inhibit secondary metabolite synthesis [32,133,137]. The carbon source serves as the vital controlling agent for secondary metabolite production in *Streptomyces*, as transcriptional activation or carbon catabolite repression (CCR) is dependent on the source and concentration of carbon [133,137,138]. Carbon from sugars like glucose, maltose, glycerol, sucrose, mannose, and xylose has been reported to interfere with the production of more than 30 types of secondary metabolites (mostly antibiotics) in *Streptomyces* spp. [137,139]. The synthesis of doxorubicin in *S. peucetius* has been impeded by the utilization of glucose and galactose as the carbon source in the culture medium [140]. Sugar carbon in the media at an industrial level leads to an increase in acidification and triggers feedback inhibition through produced intermediates.

Enhanced production of DOX (1100 mg/L) was achieved by mutation treatment (UV and ART-plasma) and soybean oil as a carbon source in *Streptomyces peucetius* SIPI-11 [141]. Oil utilization has also benefited from imparting the activity as an antifoam at the industrial fermentation scale. The breakdown of oils supports the activity of malonyl Co-A and Acetyl Co-A, which are essential for the biosynthesis of secondary metabolites. Thus, employing an oil-based carbon source instead of sugar in combination with optimized fermentation conditions and selection would enhance DNR/DOX production.

### 8.2. Nitrogen Source

Nitrogen in the form of ammonia is mainly preferred by microorganisms, and the genera *Streptomyces* naturally possess a constant nitrogen acquisition by assimilating ammonia through glutamate dehydrogenase in ammonia-rich conditions and glutamine synthetase pathways in ammonia-deficient conditions [133,139,142]. The influence of various regulatory mechanisms of nitrogen in *Streptomyces* has been clearly reviewed in [143]. The forms or sources of nitrogen, like ammonium, nitrate, amino acids, and polyamines, positively impact the production of secondary metabolites in *Streptomycetes* [139,143]. Specific nitrogen sources like soy (grits, flour, peptone) and beef extract were employed to quantify the yield of different metabolites and their precursor compounds in *Streptomyces* spp. [144].

A well-established culture media, including all these macro components together with the essential microelements like Fe, Ca, Zn, S, etc., enhances secondary metabolite yield. Optimization and standardization of culture media, considering pH, combinations of nutrients, agitation, and temperature, have enhanced daunomycin production in *Streptomyces* spp. [36,141]

## 9. Engineering Culture Media—In Prospect of Improved DNR Production

Over the past decades, genetic alterations have been frequently used to enhance the production of metabolites in *Streptomyces*, improving regulatory gene expression, modifying resistance, developing efflux mechanisms, and possible combinations with strain development. However, modifications to the culture media can also potentially improve production yields. A considerable amount of research is lacking in this area, but strategies employed for other polyketide synthesis in *Streptomyces* relevant to daunomycin can provide promising insights into the enhancement of production devoid of complex and expensive gene-editing methods.

The prominent effect of DOX/DNR is its autotoxicity by intercalating with the DNA in the producers when the concentration increases. The prospective idea of this article is to prepare culture media for cultivating *Streptomyces* sp. based on binding DNR with Iron or reduced forms of iron after effluxing from the producer. The iron or iron–DNR complex is encapsulated by the oleic acid or lipid micelle layers in the culture medium, converting the DNR to inactive forms and settling with the DNR–iron–oil complex. Therefore, this hypothesis can safeguard the producer strain from toxicity and avoid inhibiting metabolite production.

### 9.1. Perturbation of Metabolite Biosynthesis in Streptomycetes

The overexpression of regulatory genes in BGCs and downregulation of repression genes and factors have always remained prominent approaches in the metabolic engineering of *Streptomyces* spp. for metabolite production [32,42,145]. On the contrary, the availability of biosynthetic precursors also serves as a critical factor generated primarily by carbon catabolism in the organisms [146,147]. Perturbation is the supply of precursors for modulating biosynthesis to improve cells’ ability to enhance secondary metabolite production. The ARCs (antibiotic remodeling compounds) screened from *Streptomyces coelicolor* A3(2) are known to stimulate metabolite production by acting as precursors [148]. The ARC2, similar to the antimicrobial compound triclosan, has been reported to inhibit fatty acid synthesis partially, utilize the acetyl CoA for polyketide biosynthesis, and improve the actinorhodin yield in *S. coelicolor* [148,149]. Using triclosan as an elicitor of polyketide biosynthesis in Streptomyces sp. has been reported to overproduce metabolites like oligomycin, salinomycin, erythromycin, and actinorhodin [147,150,151].

### 9.2. Media Construction for Three-Way Interaction (DNR–Iron–Oligolipid)

The achievement of a prospective three-way interaction from can be achieved from distinctive methods under a single hood with critical optimization of conditions like pH, temperature, pressure, and incubation time, and initial components like natural chelators, metal salts, and nutrient sources. The biosynthesis of FeO and Fe ion particles from their salts like FeCl_3_ using phytoextracts has been employed in nanoparticle synthesis over decades [152,153]. The phytic acid present in plants, cereals, and legumes has a tremendous metal chelation potential [154]. The phytate–metal complex is stable and cannot be liberated in wide pH ranges. Phytates from soybean or soy-derived products have a high iron-binding ability, which is considered a major drawback in diet and nutrition [155,156]. Thus, utilizing soybean phytates in the culture medium facilitates iron binding and chelation.

As discussed in the above carbon sources section, the oil source of carbon in the culture media for *Streptomyces* describes its prominence in improved production in several instances, including erythromycin [157], clavulanic acid [158], doxorubicin [159], salinomycin [160], and josamycin [161]. Employing crude oils, including the raw plant parts with phytic acid contents, will deliver the nutrient carbon source and act as a reducing agent for iron in the media. Crude oils of soybean and pomace have enhanced clavulanic acid production in *Streptomyces*, which is also a waste-to-value strategy [158,162]. The crude plant oil substrate used for the cultivation media forms micelles due to elevated temperature and pressure during autoclaving and encapsulation of Fe^2+^/Fe^3+^ particles. After inoculation of the perturbated *Streptomyces ceoruleorubidus* culture to the cultivation media, the production of daunorubicin takes place and is effluxed out into the cultivation media.

Considering the lipophilic nature of daunorubicin, the produced, effluxed DNR into the medium can interact with the oligolipid surface layer with Fe ion particles from the oil-based medium [124]. The interaction between anthracycline and metal ions, especially iron, has the potential to form complexes that demonstrate high stability constants in the medium [163]. The produced and effluxed DNR by the Streptomyces strain interacts directly with the Fe–micelle to form a DNR–Fe–micelle complex [86,123,164]. Thus, the catchment of the produced metabolite in an inactive form helps in evasion of the toxicity to the producer organism.

Streptomycetes are also well known for their metal resistance, which involves their intricate intracellular iron homeostasis mechanisms [165]. As a defensive strategy, the reduced iron entering the cells is segregated and secreted externally through ABC pumps. The defensive strategy of *Streptomyces* in effluxing the excess DNR re-initiates the production of new DNR molecules inside the cells, resulting in improved productivity. Therefore, the enhancement of the production of daunomycin in *Streptomyces* using this media construction approach can be established with reduced costs and negligible metabolic engineering of strains.

Moreover, the complex of iron and anthracyclines is known to be less cardiotoxic than its original counterparts [119,122]. A similar interaction has been reported in Adriamycin: iron complex with phosphatidylcholine in the presence of oxygen to form a compound similar to cardiolipin [166]. The liposome-associated doxorubicin was reported to have reduced systemic and cardiotoxicity in clinical trials for humans and mice [127,128,129]. Thus, the three-way compound can also be employed for a liposomal drug delivery approach after extensive trials. Additional investigations in this field could result in more streamlined and economically feasible techniques for manufacturing daunomycin and other crucial secondary metabolites.

## 10. Conclusions

The authors conclude that this review is fabricated with the aim to use a traditional method of switching cultivation media sugar to the oil-based one and develop the *Streptomyces ceoruleorubidus* in a contemporary way to favor the active usage of lipids as a source of energy and entrap the produced DNR/DOX with iron present in the medium. This approach would enhance the production of DNR/DOX by the strain, and the produced metabolite does not interact with the producing strain and evades self-toxicity. However, over the past decades, the efforts of various groups working with the enhancement of daunomycin and doxorubicin production have involved the engineering of the strain (gene regulation, resistance genes development, metabolic pathway regulation) and use of sugar-based media; our prospective approach sheds light on the topic in a different approach which remains the first report in the context of daunomycin production. Adapting this strategy would improve secondary metabolite yield and benefit the extraction of the derived compound (DNR/DOX) by reducing the expenses at a large-scale fermentation.

## Data Availability

No new data were created or analyzed in this study.
